# Correlations and Kappa Distributions: Numerical Experiment with 3D Collisions and Debye-like Shielding

**DOI:** 10.3390/e28060688

**Published:** 2026-06-14

**Authors:** David J. McComas, George Livadiotis, Nicholas Sarlis

**Affiliations:** 1 Department of Astrophysical Sciences, Princeton University, Princeton, NJ 08540, USA; glivadiotis@princeton.edu (G.L.); ns7888@princeton.edu (N.S.); 2 Physics Department, National and Kapodistrian University of Athens, Panepistimiopolis, 15784 Athens, Greece

**Keywords:** space plasmas, kappa distributions, heliosphere, correlations, numerical experiment

## Abstract

Contrary to the common assumption of Maxwell–Boltzmann (MB) distributions, space plasmas are characterized by kappa distributions and reside in thermodynamic stationary states out of classical thermal equilibrium, owing to the correlations between the charged plasma particles. In this study, we extend prior work to include realistic 3D collisions and Debye-like shielding of the correlations to show how these two processes compete in the development of realistic plasma particle velocity distributions. We modify our prior numerical experiment to incorporate both 3D collisions and correlations that include realistic Debye-like shielding of plasma particles and run it over many collisions until it becomes stationary. While 3D collisions alone produce Maxwell–Boltzmann (MB) distributions of the particles (*κ* → ∞), introducing correlations drives the distributions to stationary states with finite thermodynamic kappa (*κ*), where stronger correlations produce lower values of *κ*, as observed in space plasmas. Further, development of correlation clusters around each collision rapidly produces thermodynamic systems where the Debye length is proportional to $\sqrt{1+1/{\kappa_{0}}_{\mathrm{th}}}$, for invariant thermal kappa ${\kappa_{0}}_{\mathrm{th}}$, just as predicted by theory. This simple numerical experiment explores much more realistic particle interactions to show how 3D collisions and properly shielded correlations compete to produce stationary states of plasma particle kappa distributions and illuminates how long-range interactions correlate particles over the scale of the Debye lengths.

## 1. Introduction

Particle distributions in space plasmas are almost always observed to be kappa distributed with relatively small values of *κ*, instead of being Maxwell–Boltzmann (MB) distributed [1,2], where *κ* ⟶ ∞. These observations began in the 1960s with the original studies of [3,4,5] and now extend from the solar wind to planetary magnetospheres, throughout the heliosheath, and even beyond the heliosphere to interstellar and astrophysical plasmas (Table 1).

Beyond their observational basis in space plasmas, kappa distributions are linked to nonextensive statistical mechanics and thermodynamics (e.g., see original papers: [97,98,99]; reviews: [100,101,102,103,104,105]; and books: [106,107]). These kappa distributions are physically founded and arise naturally from the thermodynamics of systems in stationary states that are not in classical thermal equilibrium [100] and in fact constitute the most generalized particle energy distributions consistent with thermodynamics [108,109].

For kappa $\kappa (d)=\kappa_{0}+\frac{1}{2}d$, with *d* kinetic degrees of freedom and invariant kappa $\kappa_{0}$ [15,73,110] the kappa velocity ***u*** distribution is given by

(1a) $$P_{u}(u;\kappa_{0})\sim {\left[1+\frac{1}{\kappa_{0}}\frac{{\left(u-u_{b}\right)}^{2}}{\theta^{2}}\right]}^{-\kappa_{0}-1-\frac{1}{2}d},P_{u}(u;\kappa_{0}\to \infty )\sim \exp\left[-\frac{{\left(u-u_{b}\right)}^{2}}{\theta^{2}}\right]$$

with bulk velocity ***u***_b_ and thermal speed *θ* (temperature *T* in speed units). As shown by (see Appendix A in [6]), in terms of kinetic energy $\varepsilon =\frac{1}{2}m{\left(u-u_{b}\right)}^{2}$, this distribution becomes

(1b) $$P_{E}(\varepsilon ;\kappa_{0})\sim {\left(1+\frac{1}{\kappa_{0}}\cdot \frac{\varepsilon }{k_{B}T}\right)}^{-\kappa_{0}-1-\frac{1}{2}d},P_{E}(\varepsilon ;\kappa_{0}\to \infty )\sim \exp\left(-\frac{\varepsilon }{k_{B}T}\right).$$

Thus, the two fundamental thermodynamic parameters of temperature and kappa are physically independent and together fully define any kappa distribution. We note that we sometimes refer to *κ* as the “thermodynamic kappa” to stress its independent physical origin and connection to thermodynamics [15,75,82,110].

Long-range interactions produce correlations [111] which reduce the thermodynamic kappa of the entire distribution [73] while varying *κ* values characterize a spectrum of stationary states in a generalized understanding of thermodynamics [102]. A generalized definition of thermal equilibrium is provided by these stationary states, where the particles have correlations amongst themselves, entropies that are not simply additive, and particle velocities and kinetic energies that are kappa distributed with *κ* < ∞ [111]. Further work [19,75,109,110,112] led to the definition and subsequent use of the “entropy defect” $S_{D}=\frac{1}{\kappa }\cdot S_{A}\cdot S_{B}$, which is the loss of entropy when assembling two correlated systems A and B (related mathematical analysis was also done by [113,114,115,116,117,118]). Analogous to the mass defect when assembling subatomic particles, the entropy defect quantifies the additional correlations developed between the particles (and thus loss of disorder or entropy) when two such systems are combined. Thus, the magnitude of the entropy defect, 1/*κ*, sets a maximum value of entropy [110,112,116,117]. Finally, when any non-stationary particle distribution evolves into a stationary state, it becomes a kappa distribution with the same kappa as derived for the initial distribution through the entropy defect [109].

Other literature suggests that kappa distributions represent just one approach to understanding and explaining “long-tail non-Maxwellian” distributions. For example, works such as [118,119] have shown that kappa distributions can be generated using Superstatistics. Moreover, Ref. [120] recently demonstrated that kappa distributions can arise for an assumed requirement of a specific dependence between the kinetic energy of a particle and its environment. Others in the community do not even accept Equation (1) for defining kappa distributions (see, for example, the books by [121,122]).

While we agree that the mathematical form of kappa distributions can be arrived at through a variety of other pathways, we believe that kappa has a far more fundamental grounding in thermodynamics and physics more broadly. In order to have temperature be a physically meaningful quantity, a system must be in a stationary state. This stationarity produces a kappa value that is defined by Equation (1), which we call the thermodynamic kappa. Moreover, kappa is even more fundamental in that 1/*κ* provides the magnitude of the Entropy Defect for systems even when they are non-stationary.

After all of the observational and theoretical work on kappa distributions and their physical origins over the past couple of decades, Ref. [6] asks the question “Is there yet another, independent way to test our understanding of kappa distributions and their origin in correlations from long range interactions?” These authors answered with the development of a first set of numerical experiments that tested and ultimately supported the thermodynamic framework of kappa distributions and their connection with correlations through long-range interactions. In particular, Ref. [6] developed a simple numerical model that produced 2D collisions with random energy sharing between numerical particles and showed that these collisions lead to MB velocity and energy distributions as expected. They then added ad hoc correlations amongst particles through “pseudo-collisions” with various amounts of predetermined energy sharing between particles and demonstrated that these produce stationary kappa distributions with finite *κ* values. They further showed that more frequent and more asymmetric energy sharing produced lower *κ* values, confirming our physical understanding of the thermodynamic kappa.

A follow-on study by [123] extended the numerical experiments and found that (1) the combination of numerical collisions and correlations produces a kappa describable analogously to an “interatomic” potential among the particles, (2) thermodynamic kappa arose even just for pairwise short-range correlations, especially in correlated clusters; and (3) stationary states do not arise from multi-particle correlations. Further, they showed that when collisions were turned off, the distributions approached “anti-equilibrium” ($\kappa_{0}$ → 0), which is the farthest possible state of a distribution from classical thermal equilibrium [71,73,102].

The Debye length, which characterizes the distance over which shielding and collective behavior occurs, has been interpreted in several different ways (e.g., Section 5.5.5 in [106] also [124,125]). Historically, the Debye length was simply defined as a characteristic length that mathematically represented the distance over which the potential energy from a charge perturbation falls to 1/e of its unshielded value [126,127]. A second, more physically motivated definition is the distance where the potential energy from a shielded charge perturbation decreases enough to equal the local thermal energy [128,129]. With this definition, shielding adjustments are effectively overwhelmed by thermal fluctuations at distances greater than the Debye length. Finally, Ref. [124] proposed that the standard deviation of the positional charge distribution could also provide a third definition of the Debye length.

Our numerical experiments combine pseudo-collisions with physical collisions; the former produce correlations, which reduce randomness, while collisions increase randomness in the system. In this study, we expand our prior work by (1) generalizing the previous 2D collisions to be fully 3D and (2) adding Debye-like correlation clusters to the particles such that correlating interactions are strongest amongst nearest particles and drop off with distance as occurs in Debye shielding. Section 2 describes the enhancements to our simple numerical experiment, while Section 3 includes Debye-like correlations and shows the results. Finally, Section 4 provides our conclusions and discussion, while Appendix A describes our methods for estimating kappa, and Appendix B documents the enhanced, but still simple computer code used in these numerical experiments.

## 2. Enhanced 3D Numerical Experiment

Following our previous numerical studies [6,123] we define a large number of particles (~10^6^) in sequential positions, *i* = 1,…, *n*, with a distribution of energies *E_i_*. Numerical collisions conserving both energy and momentum occur between randomly chosen particles *i* and *i* + 1. Our earlier studies included 2D (planar) collisions with the single degree of freedom of a variable ratio of the post-collision particle energies (conserving the total energy). Over many iterations, that procedure led to 2D velocity distributions, which in the absence of correlations naturally generated MB distributions, regardless of the initial distributions.

In this study we include 3D collisions to produce fully 3D velocity distributions. We do this by assuming ‘hard’ sphere collisions between two particles, labeled “1” and “2”, respectively. We introduce two angles $\theta_{1}$ and $\theta_{2}$ corresponding to the polar angles (in spherical coordinates) of their momenta ***J*_1_** and ***J*_2_** with respect to the line passing through the centers of the two ‘hard’ spheres at the point of contact. Energy and momentum conservation lead to the following expressions for the new momenta ***J*′_1_** and ***J*′_2_** after their collision:

(2a) $${J^{\prime }}_{1}=\sqrt{{\left(J_{1}sin{\;\theta }_{1}\right)}^{2}+{\left(J_{2}\cos{\;\theta }_{2}\right)}^{2}},$$

(2b) $${J^{\prime }}_{2}=\sqrt{{\left(J_{1}cos{\;\theta }_{1}\right)}^{2}+{\left(J_{2}\sin{\;\theta }_{2}\right)}^{2}.}$$

If ${\cos\theta }_{1}$ and ${\cos\theta }_{2}\;are$ uniformly distributed within the interval [−1, 1] we obtain a 3D collision. We note that while hard sphere collisions differ from Coulomb collisions as occur in plasmas, they do a good job of mimicking the accumulated effect of many small-angle Coulomb collisions in efficiently randomizing the particle energies.

We incorporated correlations in our prior numerical experiments by sharing momentum between a different, randomly drawn *j* and *j* + 1 pair, but with a prescribed sharing of the particle momenta. For each, we conserved total energy but took a fraction *f* of that energy of each donor (0 ≤ *f* ≤ 1) and transferred it to the adjacent recipient. Because *f* is predetermined in this procedure, there are no degrees of freedom, so this procedure generates correlations through controlled or “pseudo-collisions” amongst the numerical particles. We enhanced correlations by transferring an increasing *f* between the two correlated particles and also by allowing *M* additional *j* pairs exchanging energies after each *i* collision. In our initial new experiment, we performed pseudo-collisions on independently selected correlation pairs of particles, *j* and *j* + 1, instead of the *i* and *i* + 1 collision pair. In the current study, we carry out the pseudo-collision on the same pair of particles, immediately after their collision. With 3D instead of 2D collisions and performing the pseudo-collisions directly on each *i* and *i* + 1 colliding pair, we produce much more realistic correlations since every real particle collision in a plasma includes both 3D thermal randomization and interparticle correlations.

We analyze the results through the probability density function (pdf) *f*(E) of the energy *E* (see Appendix A in [97]), for which, as usual, *f*(*E*)∙d*E* provides the probability that the energy lies in the range from *E* to *E* + d*E*. For the current study, we extend the 2D results to 3D by employing the density of kappa distributions, see Equation (1b), with invariant kappa *κ*_0_. The pdf is then just $P_{E}\left(E;\kappa_{0}\right)$ multiplied by the density of states g(E) for the *d*-dimensional space, $g(E)\sim E^{\frac{1}{2}d-1}$. Thus, we obtain

(3) $$f\left(E\right)=N\cdot E^{\frac{1}{2}d-1}{\cdot \left(1+\frac{1}{\kappa_{0}}\cdot \frac{E}{k_{B}T}\right)}^{-\kappa_{0}-1-\frac{1}{2}d},$$

which for *d* = 3 simplifies to

(4) $$f\left(E\right)={N\cdot E^{\frac{1}{2}}\cdot \left(1+\frac{1}{\kappa_{0}}\cdot \frac{E}{k_{B}T}\right)}^{-\kappa_{0}-\frac{5}{2}},$$

where *N* is the normalization constant that ensures $\int_{0}^{\infty }f\left(E\right)dE=1$.

**Figure 1:**
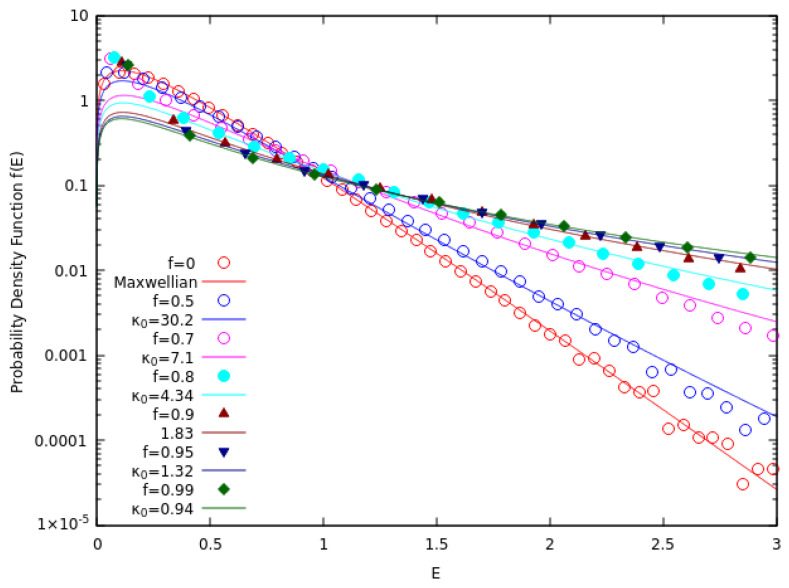
Particle energy pdf plotted as a function of normalized energy for *n* = 10^6^ particles after 3 × 10^5^ Monte Carlo Steps (MCS), and the cases of *f* = 0 (red circles, fitted by Equation (5) shown by the red line), *f* = 0.5 (blue), *f* = 0.7 (magenta), *f* = 0.8 (cyan), *f* = 0.9 (brown), *f* = 0.95 (dark-blue), *f* = 0.99 (green). The solid lines of the same colors are pdfs estimated by Equation (4) for various values of $\kappa_{0}$ (see Appendix A for the method of determination).

Figure 1 shows the 3D pdf of particle energy for a single pseudo-collision per collision and *f* = 0, 0.5, 0.7, 0.8, 0.9, 0.95, and 0.99. When *f* = 0, there are no correlations, the distribution of momentum *f*(*J*) is given by a MB (*κ* ⟶ ∞), while the respective pdf for energy *E*, given by

(5) $$f\left(E\right)=N\cdot E^{\frac{1}{2}}\cdot \exp\left(-\frac{E}{k_{B}T}\right),$$

is tail-dominated due to the exponential term (see the red line in Figure 1). For all values of the fraction *f* greater than zero, the distribution becomes a kappa distribution with a finite $\kappa_{0}$ that decreases monotonically for larger and larger values of *f* (see Appendix A for the method we use to estimate kappa). As in our prior two studies, deviations of the simulated distributions from a kappa distribution at the highest energies are caused by limitations of the finite number of particles *N* and iterations *τ* used in the numerical experiment and the *κ*-like behavior extends to higher energies as *N* and *τ* increase. We note that these results do an excellent job of both (1) approaching power-law high-energy tails [15,81] and (2) replicating the low-energy, non-power-law portion of the kappa distributions.

We note that the modulus of momentum *p* = $\left(≝\left|\vec{p}\right|\right)$ is initially considered to be a uniformly distributed random variable in the region [0, 1]. Thus, the probability density function $f\left(p\right)$ of the momentum p is simply set to $f\left(p\right)=1$; this includes the density of states, i.e., $\int_{0}^{1}f\left(p\right)dp=1$. The kinetic energy is $E=p^{2}$ (i.e., for simplicity we have set 2m=1). Hence, the temperature given by the average value of energy is $\langle E\rangle =\langle p^{2}\rangle =\int_{0}^{1}p^{2}f\left(p\right)dp=\int_{0}^{1}p^{2}dp={\left[\frac{p^{3}}{3}\right]}_{0}^{1}=\frac{1}{3}$. That is, the temperature in our numerical experiment is $k_{B}$*T* = $\frac{\langle E\rangle }{\frac{3}{2}}=\frac{2}{9}$ and since energy is conserved in every collision and pseudo-collision, temperature is also conserved. Thus, while the kappa values that correspond to various levels of correlation between the particles varies with *f*, the average temperature does not.

On a microscale, Debye shielding arises from the response of charged particles (especially highly mobile electrons) to changes in other charged particles’ positions, and thus in their inter-particle electrostatic forces. Because the nearest particles to any given particle are affected first and most strongly by some change, they respond and thereby produce some electrostatic shielding that reduces the impact of the initial change on particles farther away. Because particle positions are constantly changing due to their thermal motions, the inter-particle forces and shielding are also constantly changing, which produces collective behavior within the population of charged particles.

**Figure 2:**
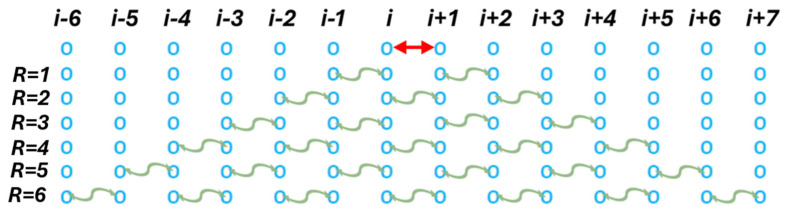
Pyramid structure of the correlation steps that includes pseudo-collisions (green arrow) between adjacent particles (blue) after an initial random collision between particles *i* and *i* + 1 (red). *R* indicates the number of layers (steps) in the correlation cluster, with *R* + 1 pseudo-collisions at each layer.

In our prior work, we sought to examine how correlations could propagate outward in through pairs of nearest neighbors. For this we [123] used a scheme similar to that shown in Figure 2. In the current study, we include 3D instead of 2D correlations and instead of starting correlations at some independent randomly drawn *j* location, we allow the correlations to spread the information about each most recent *i* and *i* + 1 collision. This is done progressively outward (and back inward toward *i* and *i* + 1) in a pyramidal structure around each collision, producing a correlation cluster. Each *R* step includes *R* + 1 nearest neighbor correlation events as shown in this figure, so by the time *R* = 6, 27 pseudo-collisions have occurred, and these have involved 14 sequential particles, some in as many as six pseudo-collisions.

**Figure 3:**
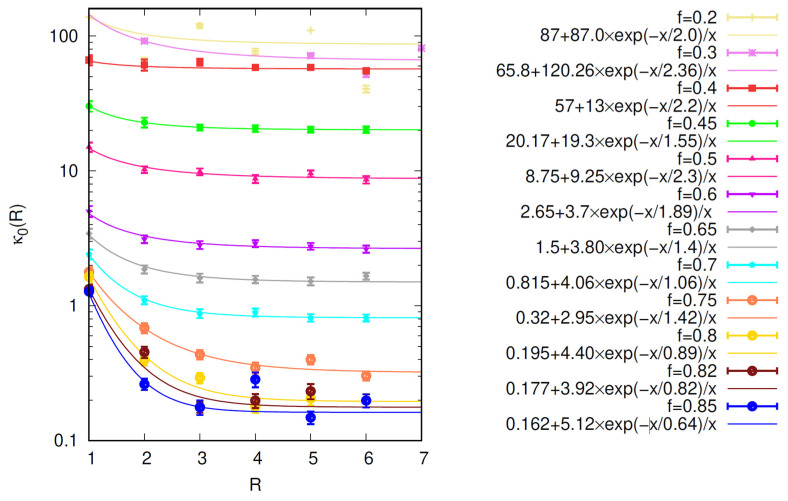
Variation in *κ*_0_ with *R* (number of pyramid steps) for values of *f* = 0.2, 0.3, 0.4, 0.45, 0.5, 0.6, 0.65, 0.7, 0.75, 0.8, 0.82, 0.85. For each *f*, we performed a least square fit to the expression $\kappa_{0}^{\infty }+A\cdot exp\;\left(-\frac{R}{\Lambda }\right)/R$ as indicated by the continuous curve of the same color.

In running the procedure shown in Figure 2, we were able to calculate $\kappa_{0}$ as a function of correlation step $\kappa_{0}(R)$ for various correlation fractions *f*. Figure 3 shows these results for a variety of *f*-values from 0.2 to 0.85 and number of correlation steps *R* = 1–7. Consistent with prior results, we found that larger *f* and *R*, produce smaller${\;\kappa }_{0}$, indicative of more correlation in the system and distributions farther from a Maxwellian.

What surprised us in reviewing the results in Figure 3 was just how quickly $\kappa_{0}\left(R\right)$ was leveling out, even with small numbers of *R* layers, and rapidly approaching specific asymptotic values for each *f* value. Thus, with proper fitting, we are able to find the asymptotic value of $\kappa_{0}$, or $\kappa_{0}^{\infty }$. This means that while the derived $\kappa_{0}^{\infty }$ is still a function of our arbitrary procedure with correlation fraction *f*, it is not a function of the extent of the propagation of the correlation information away from each random collision, and is thus essentially independent of the correlation procedure that we invented. The last is an outcome of thermodynamics; once the thermodynamic equilibrium is achieved, the correlations in stationary states are those associated with kappa distributions [109,111], independent of the initial or eventual forms and origins of the correlations.

## 3. Kappa, Level of Correlation, and Connection to Debye-like Shielding

The value of thermodynamic kappa ${\kappa_{0}}_{\mathrm{th}}$ is related to the average potential energy, normalized to the thermal energy [106,130]

(6) $${\kappa_{0}}_{\mathrm{th}}\sim \frac{\left\langle \Phi (\vec{r})\right\rangle }{k_{B}T}.$$

We note that this equation also includes a dependence on the polytropic index; however, this is unimportant for our discussion here, and one may choose isobaric process for which this dependence vanishes.

While the thermodynamic value of kappa is a macroscopic observable, a radial profile of this parameter, $\kappa_{0}(\vec{r})$, is possible within a Debye sphere where there are also statistical variations. Following the connection between statistical mechanics and thermodynamics, the positional average of $\kappa_{0}(\vec{r})$ gives its thermodynamic value; therefore, we expect that the radial profile of kappa would follow that of potential,

(7) $$\kappa_{0}(\vec{r})\sim \frac{\Phi (\vec{r})}{k_{B}T}.$$

Far from the potential’s center, where it drops to zero, the value of kappa recovers its thermodynamic value, that is,

(8) $$\kappa_{0}(\vec{r})={\kappa_{0}}_{\mathrm{th}}+A\cdot \frac{\Phi (\vec{r})}{k_{B}T}.$$

Next, we consider plasmas with 3D spherical symmetry, so that

(9) $$\kappa_{0}(r)={\kappa_{0}}_{\mathrm{th}}+A\cdot \frac{\Phi (r)}{k_{B}T}.$$

Here we use the Debye potential, $\Phi (r)=A\cdot e^{-r/\lambda_{D}}/r$, for some coefficient *A* and use the second definition of Debye length described above—that where the potential energy becomes equal to the thermal energy, i.e., $\Phi (r)=ek_{B}T\lambda_{D}\cdot e^{-r/\lambda_{D}}/r$. Hence, Equation (9) becomes

(10) $$\kappa_{0}(r)={\kappa_{0}}_{\mathrm{th}}+e\lambda_{D}\cdot e^{-r/\lambda_{D}}/r,$$

Note that this expression can be generalized for any spatial dimensionality, i.e.,

(11) $$\kappa_{0}(r)={\kappa_{0}}_{\mathrm{th}}+e^{1-r/\lambda_{D}}\cdot {\left(r/\lambda_{D}\right)}^{-\frac{d-1}{2}},$$

(see. Ref. [124] for details).

In our numerical experiment, the value of kappa, $\kappa_{0}(R)$, decreases with increasing correlation level *R*. This expression appears to be exponential and described by $e^{-R/\Lambda }$, because it is approximately linear on a semi-log scale. Moreover, at *R* = 0 there are only random collisions without any pseudo-collision steps, so kappa at *R* = 0 is infinity (Maxwellian). This behavior can be modeled with a pole at zero, namely, $e^{-R/\Lambda }/R^{a}$. Moreover, at *R* → ∞, the value of kappa does not tend to zero but to its average value, that is, the macroscopic or thermodynamic observable. Therefore, we end up with the expression

(12) $$\kappa_{0}(R)={\kappa_{0}}_{\mathrm{th}}+A\cdot e^{-R/\Lambda }\cdot R^{-a},$$

which is identical to the theoretical relation in Equation (11). The numerical experiment mimics the local positional profile of kappa, as well as the asymptotic convergence of kappa towards its constant thermodynamic value, $\kappa_{0}^{\infty }⟶{\kappa_{0}}_{\mathrm{th}}$; (hereafter we use this notation of thermodynamic kappa and for the asymptotic value of Equation (12)).

**Figure 4:**
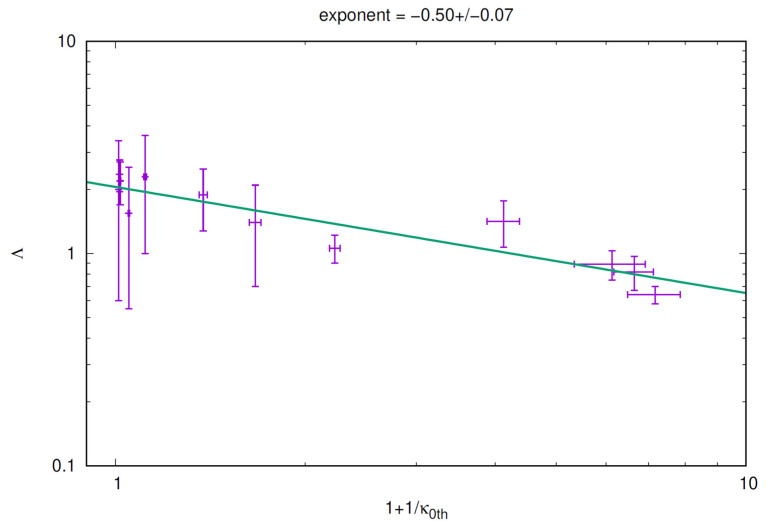
Variation in Λ as a function of the quantity 1 + 1/${\kappa_{0}}_{\mathrm{th}}$ from Figure 3. We find a power-law relation of 2.06/$\sqrt{1+1/{\kappa_{0}}_{\mathrm{th}}}$ by fitting the data points with their respective errors in this log-log diagram.

To examine this relationship in more detail, Figure 4 shows Λ as a function of 1 + 1/${\kappa_{0}}_{\mathrm{th}}\;$for all of the different values of *f* shown in Figure 3. By fitting a line in the log-log plot, we find a simple power law with an exponent of −0.50 +/−0.07 (green line).

The results of the numerical experiment are well described by Equation (12). Moreover, in order to show that the parameter Λ describes the physical parameter of the Debye length λ_D_, we examine the connection between the observed values of Λ and ${\kappa_{0}}_{\mathrm{th}}$. Theoretically, their expression should be

(13) $$\Lambda =\Lambda_{\infty }\cdot {\left(\frac{{\kappa_{0}}_{\mathrm{th}}}{{\kappa_{0}}_{\mathrm{th}}+1}\right)}^{\frac{1}{2}}.$$

or

(14) $$\Lambda =\Lambda_{\infty }\cdot {\left(1+\frac{1}{{\kappa_{0}}_{\mathrm{th}}}\right)}^{-\frac{1}{2}}.$$

**Figure 5:**
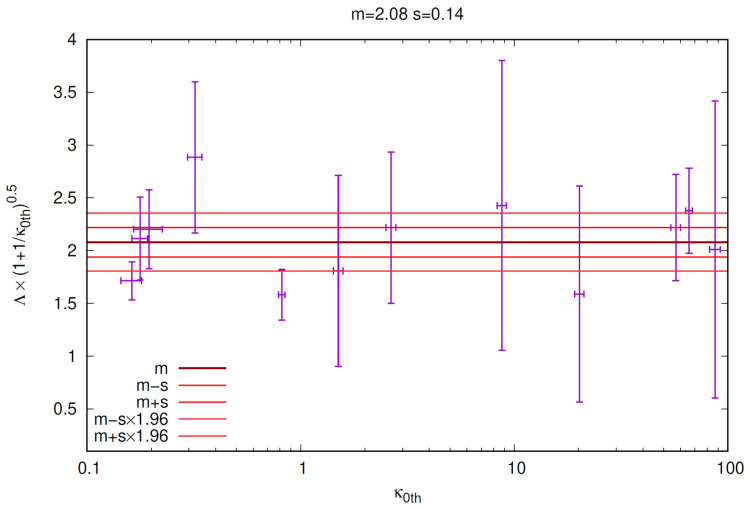
Values of Λ$\cdot \sqrt{1+1/{\kappa_{0}}_{\mathrm{th}}}$, corresponding to Λ_∞_, as a function of ${\kappa_{0}}_{\mathrm{th}}$. These results are consistent with a single, constant value for Λ_∞_ of 2.08+/−0.14.

Figure 5 shows how the parameter Λ$\cdot \sqrt{1+1/{\kappa_{0}}_{\mathrm{th}}}$ (corresponding to Λ_∞_) varies for different values of ${\kappa_{0}}_{\mathrm{th}}$ from our multiple numerical experiment runs with differing values of the fraction *f*. We find no systematic or statistically significant variations and thus conclude that a single, constant value Λ_∞_ describes this relationship. The mean value we find is ~2.08 with a standard error δΛ_∞_ = 0.14. The additional horizontal lines depict the ± δΛ_∞_ and the 95% confidence intervals; clearly the value is fully consistent with the theoretical value of 2.0.

**Figure 6:**
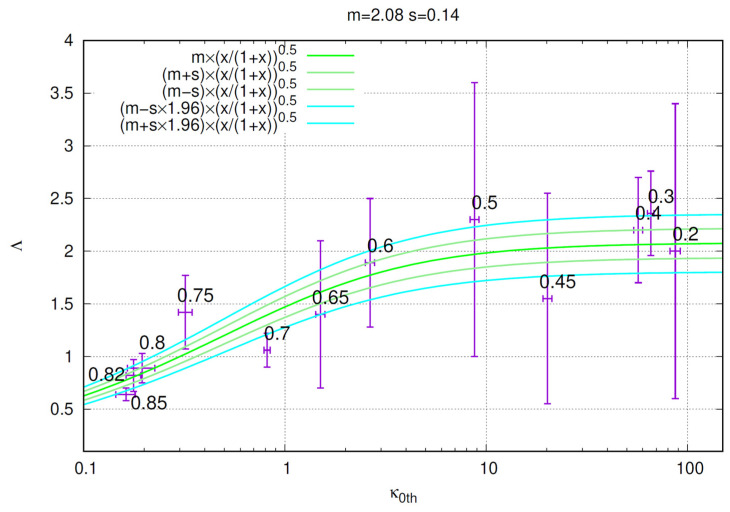
Summary figure depicting Λ (= λ_D_) versus$\;{\kappa_{0}}_{\mathrm{th}}$together with the Λ_∞_ ± δΛ_∞_ and the 95% confidence intervals resulting from the expression Λ = Λ_∞_/$\sqrt{1+1/{\kappa_{0}}_{\mathrm{th}}}$.

Through our numerical experiment, we have shown that (a) the nonlinear fitting between $\left(X\equiv 1+1/{\kappa_{0}}_{\mathrm{th}},Y\equiv \Lambda \right)$ is ~0.5; (b) the values of $\Lambda \cdot {\left(1+1/{\kappa_{0}}_{\mathrm{th}}\right)}^{\frac{1}{2}}$ can be fitted by a constant (=$\Lambda_{\infty }$); and (c) the nonlinear fit of Equation (8) shows concavity of Λ as a function of ${\kappa_{0}}_{\mathrm{th}}$. Figure 6 summarizes these results by plotting the relationship of Λ (=λ_D_) as a function of ${\kappa_{0}}_{\mathrm{th}}$. The central dark green curve shows the best fit function; other curves show this function +/− standard errors and again the +/−95% confidence levels.

## 4. Conclusions and Discussion

This study extends our prior numerical experiments [6,123] to include full 3D collisions instead of the prior 2D collisions. This development is important because it removes any potential issues or lingering concerns that somehow the dimensionality of the system could be affecting our results in some not-understood way. We also changed the procedure to implement the correlating pseudo-collisions to occur in a correlation cluster around the same *i* and *i* + 1 particles as the prior collision because all real collisions between particles in space plasmas have both randomizing and correlating aspects. Along the way, we also needed to develop and implement an improved procedure for determining small values of $\kappa_{0}$ (Appendix A). With these much higher fidelity calculations, we were able to generate results that closely emulate real plasmas (or any systems with such correlations).

With these improvements, we revalidated our prior results. In particular, we showed definitively that stationary states are described by kappa distributions, where correlations and collisions compete. As collisions become increasingly dominant over correlations, the thermodynamic kappa tends to infinity and MB distributions are produced; in contrast, as correlations increasingly dominate over collisions, the thermodynamic kappa tends to its lowest limit (*κ*_0_ ⟶ 0), and the number of correlated particles approaches the whole number of particles.

A new and even more important result of this current work is that correlation clusters developed around collisions emulate Debye-like shielding and produce kappa values that rapidly approach their asymptotic $\kappa_{0}$ values. We showed that after only 6 steps $\left(R\right)$ of progressively widening correlation sharing around an *i* and *i* + 1 collision pair (see Figure 2) the asymptotic behavior and values could already be derived. This allowed us to draw much more solid conclusions as the details of our arbitrary procedure for sharing correlation information through correlation fraction *f*, and number of steps $\left(R\right)$ are no longer critical. Instead, because once the system achieves thermodynamic equilibrium, correlations in stationary states are associated with kappa distributions and independent of the origins or final forms of the correlations. Thus, our results are essentially independent of the ad hoc correlation procedure that we invented and fully consistent with thermodynamics.

We end by noting that much of the recent explosion in understanding of kappa distributions of particles in stationary states, the thermodynamic *κ* that persists even when the distributions are non-stationary, and the powerful concept of the Entropy Defect, have been driven by the study of space plasmas. This is in part because kappa values decrease instead of increasing in the solar wind beyond ~10 au, owing to the addition of pickup ions and their highly ordered motion (low dimensionality), which becomes shared with the core solar wind. This process reduces the entropy and generates smaller values of thermodynamic *κ*. The addition of PUIs persists out to the termination shock, where they become heated (increase in *T*, not *κ*) and observed as Energetic Neutral Atoms (ENAs) emanating from the heliosheath and propagating back in, where they are imaged by the Interstellar Boundary Explorer (IBEX) mission [76,131] from Earth orbit. In the heliosheath, *κ* values are especially low [78,79,80,81] and in the “far-equilibrium” region [71,72,73], approaching anti-equilibrium. The Interstellar Mapping and Acceleration Probe (IMAP) mission [132,133] launched 24 September 2025, and is now providing significantly enhanced sensitivity, resolution, and energy range for measuring these ENAs. Therefore, IMAP is now providing even more precise observations to test our physical understanding, theory, and numerical models, such as the realistic 3D collision and Debye-like shielding one developed in this study.

## Figures and Tables

**Table 1:** Example observations of kappa distributions ubiquitously in space plasmas (modified from [6]).

**1. Inner Heliosphere**	
solar corona	[7,8,9,10]
solar energetic particles	[11,12,13,14,15,16,17]
solar flares	[18,19,20,21]
solar radio emission	[22,23,24]
solar spectra	[25,26]
solar wind	[27,28,29,30,31,32,33,34,35,36,37,38,39,40,41]
corotating interaction regions	[42]
**2. Planetary Magnetospheres**	
magnetosheath	[43,44]
magnetopause	[45]
magnetotail	[46]
ring current	[47]
plasma sheet	[48,49,50]
magnetospheric substorms	[51]
aurorae	[52]
magnetospheres of giant planets, such as:	
- Martian	[53]
- Venerian	[54]
- Jovian	[55,56]
- Saturnian	[57,58,59,60]
- Uranian	[61]
magnetospheres of planetary moons, such as:	
- Io	[62]
- Europa	[63]
- Enceladus	[64]
cometary magnetospheres	[65,66,67]
**3. Outer Heliosphere and Astrophysical Plasmas**	
inner heliosheath	[68,69,70,71,72,73,74,75,76,77,78,79,80,81,82,83,84,85,86]
H-II regions	[87]
planetary nebulae	[87,88,89,90]
active galactic nuclei	[91,92]
galactic jets	[93]
supernovae	[94]
cosmological scale phenomena	[95,96]

**Figure A1:**
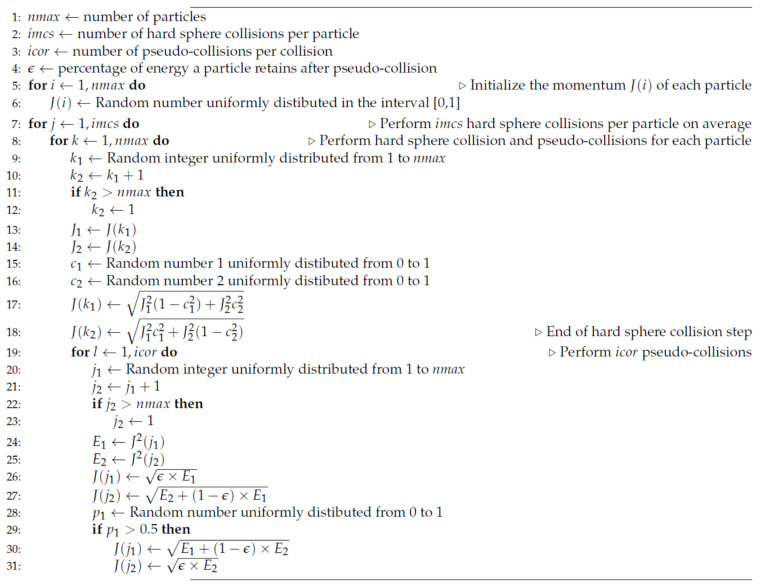
The code for the numerical experiment with 3D hard sphere collisions and pseudo-collisions.

## Data Availability

The data presented in this study are available on request from the corresponding author.

## Appendix A. Method for Estimating Kappa Using the Variation Technique

Here we present the method for finding the value of thermodynamic kappa that parameterizes the kappa distributions by using the technique of variation. This technique was first introduced by [15]. Briefly, instead of fitting the energy distribution function *f*(*E*;*κ*_0_) to find the (fitting) parameter *κ*_0_, we solve for the value of the kappa parameter for each data point and then average the produced values of kappa. This method is somewhat more complicated because there are three unknown parameters to be determined: the density, temperature, and kappa. We use this approach because not all the available particle energies contribute to the stationary state (the converging kappa distribution), which is confined to energies larger than *E* ~ 0.1 because a small fraction of the particles aggregate at lower energies, owing to the special way we construct the particle correlations [6]. Therefore, neither the density nor temperature of kappa distributions is fixed to their initial values and thus need to be determined.

In order to determine the values of the three unknown parameters, we must construct a number of *N*∙(*N*−1)∙(*N*−2)/6 combinations of 3-point data subsets, where each triplet is suitable for estimating a single value of each of the parameters. In fact, we are only interested in the value of kappa, so we proceed as follows. We first consider the quantity *X* = ln(*E*) that transforms Equation (4) to the pdf $g\left(X\right)$ of *X*:

(A1) $$g\left(X\right)={N\cdot e^{1.5X}\left(1+\frac{1}{\kappa_{0}}\cdot \frac{e^{X}}{k_{B}T}\right)}^{-\kappa_{0}-\frac{5}{2}}.$$

Then, we employ the natural logarithm of $g\left(X\right)$ denoted by $\Psi =\ln\left[g(X)\right]$ and eliminate *N* and $k_{B}T$ using a triplet of points $X_{i},\;X_{j},\;$and $X_{m}$ at which we calculate (numerically) the corresponding in the log space pdfs, i.e., $\Psi_{i}\left\{=\ln\left[g(X_{i})\right]\right\},\;\Psi_{j}\left\{=\ln\left[g(X_{j})\right]\right\},\;$and $\Psi_{m}\left\{=\ln\left[g(X_{m})\right]\right\}$. This leads to the quantity

(A2) $$f_{ijm}=\left|\frac{exp\left\{-\frac{1}{\kappa +1}\left[\Psi_{\iota }-\Psi_{j}-\frac{3}{2}\left(X_{i}-X_{j}\right)\right]\right\}-1}{e^{X_{i}}-e^{X_{j}}}-\frac{exp\left\{-\frac{1}{\kappa +1}\left[\Psi_{m}-\Psi_{j}-\frac{3}{2}\left(X_{m}-X_{j}\right)\right]\right\}-1}{e^{X_{m}}-e^{X_{j}}}\right|,$$

where κ equals $\kappa_{0}$ + 3/2, which should be minimized for the value of $\kappa_{0}$ that best describes the numerical results obtained for the energies *E*. Thus, we selected the range of X∈[−1,1], obtained the histogram of the numerical experiment results for *E* by using the TISEAN 3.0.0 package [134,135], and then found the values of κ that minimizes $f_{ijm}$ for different values of *i*, *j*, and *m*.

Having derived the values of kappa, we estimate their mean by averaging their corresponding correlation coefficients. For this, we inverted these values (1/κ) to obtain a quantity proportional to the physically significant correlation *ρ* = 1.5/$\left(\kappa_{0}+3/2\right)$ [73,136,137] see also: [138,139]) and obtained $\kappa_{0}$ and its standard error δ$\kappa_{0}$ from the mean and standard error of *ρ*/1.5. This way we obtain reliable $\kappa_{0}$ values, as shown in Figure 1 and Figure 3 of the main text.

## Appendix B. Numerical Experiment Computer Code

The computer code used for our numerical experiments is shown in Figure A1. We consider a large number of particles, *i* = 1,2,…, *N* (with *N* equal to one million for this study as the variable *nmax*) and the corresponding particle momenta {*J_i_*}*_i_*_=1,2,…,_ *_N_*. The latter are originally uniformly distributed in the region [0, 1], see line 6 of Figure A1. The specific selection of the original distribution does not affect the final stable distribution. We also assume periodic boundary conditions, which means that the particle next to the *N*-th particle is the one with *i* = 1 (see lines 11,12 and 22, 23 in Figure A1). As mentioned in the main text, the numerical experiment consists of two steps:

1. The hard sphere collision step where we randomly select a particle, *i* = *k*_1_ and collide it with the *k*_1_ + 1 particle using Equations (2a) and (2b) of the main text (see lines 13 to 18).
2. The pseudo-collision step where we randomly select a “particle” say with index *j* = *j*_1_ and give a fraction *f* (=1 − *ϵ*, see lines 21 to 31 in Figure A1) of its energy to the particle with index *j*_1_ + 1. For symmetry reasons, with equal probability (50%), the particle with index *j*_1_ + 1 instead gives the fraction *f* of its energy to the *j*_1_ particle.

Generally, such a pseudo-collision step may be repeated *icor* times per hard sphere collision; see line 18 of Figure A1.

For the description of Debye shielding the pseudo-collisions are made only between the particles connected by the green curly arrows in Figure 2 starting from the top and moving down each time we complete all the pseudo-collisions in a given line.
